# Surfactin Like Broad Spectrum Antimicrobial Lipopeptide Co-produced With Sublancin From *Bacillus subtilis* Strain A52: Dual Reservoir of Bioactives

**DOI:** 10.3389/fmicb.2020.01167

**Published:** 2020-06-11

**Authors:** Deepika Sharma, Shelley Sardul Singh, Piyush Baindara, Shikha Sharma, Neeraj Khatri, Vishakha Grover, Prabhu B. Patil, Suresh Korpole

**Affiliations:** ^1^Council of Scientific and Industrial Research (CSIR) – Institute of Microbial Technology, Chandigarh, India; ^2^Dr. Harvansh Singh Judge Institute of Dental Sciences & Hospital, Panjab University, Chandigarh, India

**Keywords:** sublancin, *B. subtilis*, *Candida*, synergistic, surfactin-like lipopeptide, emulgel

## Abstract

An antimicrobial substance producing strain designated as A52 was isolated from a marine sediment sample and identified as *Bacillus* sp., based on 16S rRNA gene sequence analysis. The ANI and dDDH analysis of the genome sequence displayed high identity with two strains of *B. subtilis* sub sp. *subtilis*. Strain A52 yielded two antimicrobial peptides (AMPs) that differed in activity spectrum. MALDI mass spectrometry analysis of HPLC purified fractions revealed mass of peptides as 3881.6 and 1061.9 Da. The antiSMASH analysis of genome sequence unraveled presence of identical biosynthetic cluster involved in production of sublancin from *B. subtilis* sub sp. *subtilis* strain 168, which yielded peptide with identical mass. The low molecular weight peptide is found to be a cyclic lipopeptide containing C_16_ β-hydroxy fatty acid that resembled surfactin-like group of biosurfactants. However, it differed in fatty acid composition and antimicrobial spectrum in comparison to other surfactins produced by strains of *B. subtilis*. It exhibited broad spectrum antibacterial activity, inhibited growth of pathogenic strains of *Candida* and filamentous fungi. Further, it exhibited hemolytic activity, but did not show phytotoxic effect in seed germination experiment. The emulgel formulation of surfactin-like lipopeptide showed antimicrobial activity *in vitro* and did not show any irritation effects in animal studies using BALB/c mice. Moreover, surfactin-like lipopeptide displayed synergistic activity with fluconazole against *Candida*, indicating its potential for external therapeutic applications.

## Introduction

Development of drug resistance is a great threat to the effective treatment of infections caused by opportunistic or nosocomial pathogens (Davies and Davies, 2010; Prestinaci et al., 2015; Li and Webster, 2018; Laws et al., 2019). Clinically, most significant drug resistant nosocomial pathogens include *Staphylococcus aureus*, *Escherichia coli, Klebsiella* spp., *Shigella* spp., *Mycobacterium tuberculosis*, *Pseudomonas aeruginosa*, *Acinetobacter baumannii*, and *Enterobacter* spp. (Alanis, 2005; Pande and Bhailume, 2014; Wong et al., 2017). *Candida* is a commensal fungus that is more often associated with infections in immunocompromised individuals. It has been reported to develop resistance to antibiotics like fluconazole, caspofungin, and amphotericin B commonly used to treat candidiasis (Eksi et al., 2013; Lum et al., 2015; Dagi et al., 2016; Ksiezopolska and Gabaldón, 2018). In 2019, Centers for Disease Control and Prevention (CDC) has included drug resistant *Candida* spp. in the list of organisms causing serious threat. In an attempt to combat such resistant pathogens, there is an urgent and compelling need to develop novel antimicrobials. To this effect, antimicrobial peptides (AMPs) including bacteriocins and lipopeptides with broad-spectrum antimicrobial activities have been found to be promising alternative candidates (Singh et al., 2014; Sharma et al., 2014; Lum et al., 2015; Raman et al., 2015; Lyu et al., 2016; Gwynne and Gallagher, 2018).

The genus *Bacillus* is a prolific producer of a wide range of antimicrobials and reported to produce peptide antimicrobial substances like bacteriocins, lipopeptides, and glycopeptides that are active against a variety of bacteria and fungi (Abriouel et al., 2011; Perez et al., 2018; Hashem et al., 2019; Miljkovic et al., 2019). According to their biosynthetic pathways, these bioactive molecules have been grouped mainly into two different classes: the first one comprises ribosomally synthesized peptides usually referred as bacteriocins and classified as ribosomally produced and post-translationally modified peptides (RiPPs) by Arnison et al. (2013). The other class comprises small antimicrobial cyclic lipopeptides synthesized enzymatically by non-ribosomal peptide synthetases (NRPS) and classified as post-ribosomal peptide synthetases (PRPS). Bacteriocins produced by *Bacillus* spp. display a high degree of target specificity against bacterial infections and also a broad spectrum of activity (Abriouel et al., 2011; Herzner et al., 2011; Ramachandran et al., 2014; Wu et al., 2019). Most of the compounds reported from *Bacillus* spp., largely belong to Class I bacteriocins that are known as lantibiotics (Klaenhammer, 1993) or lanthipeptides (Arnison et al., 2013). Sublancin is one of such lanthipeptide that has an S-linked glycocin produced by *B. subtilis* strain 168. It exhibits antimicrobial activity only against Gram-positive bacteria, including methicillin-resistant *Staphylococcus aureus* (MRSA) (Ren et al., 2018; Wang et al., 2018). Other strains of *B. subtilis* were reported to produces lipopeptides mostly pertaining to surfactins, iturins, and fengycins (Zhang and Sun, 2018). However, they differed in antimicrobial activity due to variations in length of fatty acid chains and/or amino acid composition (Mondol et al., 2013; Meena and Kanwar, 2015).

Marine ecosystem is a reservoir of multiple bacterial strains with ability to produce various bioactive compounds for survival (Debbab et al., 2010; Reen et al., 2015; Gupta et al., 2019; Paulsen et al., 2019). The genus *Bacillus* is a predominant microbial flora in marine ecosystem that differed with terrestrial strains in production of diverse classes of AMPs from species like *B. subtilis* (Ivanova et al., 1999; Berrue et al., 2009; Jin et al., 2012; Mondol et al., 2013; Tareq et al., 2014; Chen et al., 2017). Due to advancement in protein purification technology and characterization of AMPs, reports on bioactive from marine bacterial sources have also increased in the recent past. In light of the above facts, we have isolated a *Bacillus* strain from a marine sediment sample and explored the strain A52 for production of antimicrobial compounds, which have been characterized in detail.

## Materials and Methods

### Strain Isolation and Identification

Strain A52 was isolated from a sediment sample obtained from marine environment (Bay of Bengal, West Bengal, India). During isolation of the bacteria on various media, a colony on nutrient agar (NA, Hi-media, India) with inhibitory zone in their surroundings was sub-cultured on NA and preserved as 20% (v/v) glycerol stocks at −80°C until further studies. All indicator strains used in this study were obtained from Microbial Type Culture Collection and Gene bank (MTCC), Chandigarh, India, and grown on recommended media. Phenotypic properties of strain A52 including morphology, physiology, and biochemical characteristics were performed using standard procedures (Smibert and Krieg, 1994). Identity of the strain was confirmed by BLAST search analysis of 16S rRNA gene sequence (Suresh et al., 2006). Further, taxonomic position of strain was determined by phylogenetic analysis of 16S rRNA gene sequence with the nearest type strains using MEGA version 7 (Kumar et al., 2016). Evolutionary distances were computed by Tamura-Nei method and expressed as units of the number of base substitutions per site to construct a neighbor-joining phylogenetic tree. The strain was further used for whole genome sequence.

### Genome Sequence and Analyses

Genomic DNA of strain A52 was extracted and libraries were prepared. Sequencing of libraries was done as described earlier (Patil et al., 2016). Quality of obtained reads was checked using FastQC (Patel and Jain, 2012). Reads obtained were assembled using CLC Genomics Workbench 7.5 (CLC bio, Aarhus, Denmark). The average nucleotide identity (ANI) of strain A52 with genome sequences of other closely related members was calculated using ANI calculator (Yoon et al., 2017). Genome to genome distance calculator (GGDC) was used for the estimation of genetic relatedness using GGDC 2.0 web server^1^ and calculations were performed by recommended formulae 2. Whole genome sequences of reference strains were downloaded from NCBI database in FASTA format. Pangenome analysis was performed using ultra-fast computational pipeline BPGA (Bacterial Pan Genome Analysis tool)^2^ for core genome analysis.

### Antimicrobial Activity of Strain A52

The antimicrobial activity was tested by well diffusion assay as mentioned earlier (Singh et al., 2014). Strain A52 was grown for 24 h in 500 ml of nutrient broth (NB, Hi-media, India) using 1000 ml flask and subsequently cells were removed by centrifugation (10,000 rpm for 15 min at 4°C). Supernatant was filtered through 0.22 μm filter (Millipore, United States) to obtain cell-free fermented broth (CFB) that was used for antimicrobial assay (50 μl) against various indicator strains and the activity was measured as diameter of inhibition zone. Test strains used were *Staphylococcus aureus* (MTCC 1430), *Bacillus subtilis* (MTCC 121), *B. firmus* (MTCC 488), *B. licheniformis* (MTCC 429), *B. thuringiensis* (MTCC 1953), *Escherichia coli* (MTCC 1610), *Micrococcus luteus* (MTCC 106), *Steptococcus pyrogens* (MTCC 1928), *S. anginosus* (MTCC 1929), *S. oralis* (MTCC 2696), *S. mutans* (MTCC 497), *Candida albicans* (MTCC 1637), *C. albicans* (MTCC 183), *C. albicans* (MTCC 227), *C. tropicalis* (MTCC 184), *C. glabrata* (MTCC 3019), *C. haemulonii* (MTCC 2766), and *C. inconsplicue* (MTCC 1074). The phytopathogenic fungi employed in this study were *Alternaria brassicicola* (MTCC 2102), *Colletotrichum acutatum* (MTCC 1037), and *Fusarium moniliforme* (MTCC 158). Clinical isolates of *Candida* were provided by National Culture Collection of Pathogenic Fungi (NCCPF), PGIMER, Chandigarh, India.

### Production and Purification of Antimicrobial Substances

Production of antimicrobial substances was pursued in NB. However, production was also tested in minimal medium [containing (g/l): disodium hydrogen phosphate, 7.9; potassium dihydrogen phosphate, 3.0; sodium chloride, 5.0; and ammonium chloride, 1.0] supplemented with 1% carbon (glucose, sucrose, glycerol, and starch) and nitrogen sources (yeast extract, ammonium chloride, and peptone). Overnight grown culture of strain A52 was inoculated to 1 L of medium and grown to late logarithmic growth phase on a rotary shaker at 30°C. Subsequently, CFB was mixed with activated Diaion HP-20 (Sigma-Aldrich, United States) resin (2%) and the antimicrobial substance was eluted in methanol. Methanol was evaporated using a rotavapour (BUCHI R-200, Switzerland) and was redissolved in Milli-Q water to offer as crude extract. CFB was also extracted by ethyl acetate solvent extraction using 1:1 ratio (Alajlani et al., 2016). Subsequently, solvent was removed on a rota vapor (BUCHI-200, Switzerland) and the extract was dissolved in minimal amount of methanol. For purification, these extracts were applied onto reverse phase high performance liquid chromatography (HPLC) (1260 Infinity, Agilent Technologies, United States) with a semi-preparative C18 column (Pursuit 10C18 250 mm × 21.2 mm). The solvent system comprised 0.12% aqueous trifluoroacetic acid (TFA, solvent A) and acetonitrile with 0.1% TFA (solvent B). The gradient of solvent B used to run the column was as follows: 0–60% for 0–45 min, 60–80% for 45–50 min, and 80–100% for 50–55 min. Elution from the column was monitored at 220 nm and the peaks of HPLC chromatogram were collected. These fractions were tested for antimicrobial activity and the active peaks were pooled through multiple runs, and solvent was evaporated. Peptides were suspended in water and used for characterization studies like thin layer chromatography (TLC), gel electrophoresis, and MALDI.

### In-Gel Activity and TLC Overlay Bioautography

Purified antimicrobial peptides were analyzed on TLC plates (Silica gel 60 F_254_, Merck, Germany) and used to determine the purity and antimicrobial activity of the compound by bioautographic assay (Zheng et al., 2005). About 50–60 μl of the peptide solution (100 μg/ml concentration) was loaded onto individual TLC silica gel plates in duplicates and were developed using a solvent system containing chloroform: methane: water (65:12:4 v/v). Subsequently, each TLC plate was dried, cut into two vertical parts, and one part of the TLC was used for direct detection of antimicrobial activity by overlaying on NA plate containing indicator strain (*S. aureus* MTCC 1430, ∼10^6^ cells/ml). After allowing for 1 h of diffusion at 4°C, the TLC strip was removed and plates were incubated for 24 h at 37°C. The other part of TLC strip (5–10 μl of the peptide solution spot) was sprayed with ninhydrin and phosphomolybdic acid as a staining reagent to detect the presence of peptides and lipid moieties, respectively. Similarly, peptides were electrophoresed on 16% Tricine-SDS-PAGE and used for in-gel activity assay as described earlier (Baindara et al., 2016). *S. aureus* MTCC 1430 (∼10^6^ cells/ml) was used as an indicator strain in assay.

### Mass Spectrometry of Peptide

HPLC purified peptides and standard surfactin (Sigma, United States) were analyzed using Matrix-Assisted Laser Desorption Ionization (MALDI) to determine intact molecular weights of the peptides. The HPLC purified peptides were re-suspended in water (1 mg/ml) and 1 μl of peptide solution was mixed with 1 μl of matrix (CHCA, 10 mg/ml) and 2.0 μl of this mixture solution was spotted onto the MALDI 100 well stainless steel sample plate, allowed to air dry, and processed for MALDI analysis (AB Sciex 5800 MALDI-time of flight, TOF/TOF). Tandem mass spectrometry (MS/MS) data were acquired at 1,000 Hz in 1 kV MS/MS mode with 2,000 laser shots/spectrum in CID mode as mentioned earlier (Singh et al., 2014). The *de novo* sequence was generated using fragmentation pattern manually.

### Collapse Drop Assay (CDA)

The surfactant property of peptide was determined using the qualitative drop-collapse assay (Youssef et al., 2004). Mineral oil (2 μl) was added to 96-well microtiter plate and equilibrated for 24 h at 37°C. After equilibration 5 μl of purified peptides (10 μg/ml) were added to the surface of the oil and drop shape was observed after 1 min using magnifying glass. Triton X-100 and water were used as positive and negative control, respectively.

### Effect of pH, Temperature, and Proteolytic Enzymes

Sensitivity of purified peptides toward temperature, pH, and non-specific proteases was confirmed by performing bioassays. The pH stability was determined by adjusting the pH 2.0–12.0 of peptide aliquots (using 10 mM HCl or NaOH) and incubating for 4 h at room temperature. Samples were neutralized to pH 7.0 before processing for antimicrobial assay. To determine temperature susceptibility, peptides were incubated at different temperatures ranging from 37 to 100°C for 1 h and 121°C for 15 min and residual antimicrobial activity was determined at RT by well diffusion assay. Proteolytic enzymes trypsin and proteinase K (Sigma, United States) were used at three different concentrations (0.1, 1.0, and 5.0 mg/ml) to observe their effect on the peptide. The enzyme solutions were prepared in 50 mM phosphate buffer (pH 7.0). All reactions were performed at 37°C for 6 h followed by deactivation of enzyme by heating the solution in boiling water for 5 min before performing the activity assay.

### Determination of Minimum Inhibitory Concentration of Peptides

The minimum inhibitory concentration (MIC) of HPLC purified peptides was evaluated by using microtiter plate dilution assay. The peptide concentration was estimated using BCA protein assay (Thermo Fisher Scientific, United States) method as described by manufacturer. Actively growing test strains (5 × 10^5^ CFU/ml) were inoculated in a 96-well plate with different concentrations of the peptides and plates were incubated for 24–48 h at 37°C (final volume in each well is 200 μl). OD was measured at 600 nm after 24 and 48 h for bacteria and yeast, respectively, using ELISA microplate reader (Thermo, United States). MIC against phytopathogenic fungi was determined using 96-well microtiter plate assay (Kaur et al., 2016). In 96-well microplates, 100 μl of fungal spore suspension (1 × 10^5^ spores/ml) prepared in potato dextrose broth (PDB, Hi-media, India) was mixed with100 μl of fresh PDB containing different concentrations of lipopeptide. Control well contained 100 μl of fungal spore suspension and 100 μl of only medium. The microplates were incubated at 25°C and readings were recorded at 600 nm after 48 h. The lowest concentration showing no growth was considered as MIC. Each test was done in triplicate and optical density (OD_600_) of peptide treated wells were compared with uninoculated wells (blank medium control).

### Time Kill Assay

Purified AMPs were used to perform time kill assay against *S. aureus* MTCC 1430 as mentioned earlier (Baindara et al., 2016). Actively growing bacterial culture (0.2–0.3 OD at 600 nm) was centrifuged (10,000 rpm for 15 min at 4°C) and washed thrice with sterile PBS buffer. The pellet was suspended in PBS and subsequently treated with 2X and 5X MIC values of purified AMPs. Upon incubation for different time intervals, cells were removed from reaction mixture by centrifugation and washed with PBS buffer. Bacterial culture without AMP treatment was used as negative control. Samples were withdrawn at the end of different treatment time points starting from 0 to 8 h, bacterial cells were pelleted, serially diluted, and cell suspensions were plated on nutrient agar plates. For each treatment, negative controls were also processed under identical conditions and plated on NA. CFU at different time intervals were recorded after overnight incubation at 37°C.

### Toxicity Testing

For hemolysis assay blood was collected from New Zealand white rabbit, centrifuged, washed three times, and resuspended in phosphate-buffered saline (PBS). Cells were counted using hemocytometer (adjusted to 2 × 10^8^ cells/ml) and treated with different concentrations of peptides (between 10 and 200 μg/ml). They were incubated in a CO_2_ incubator for 24 h at 37°C and processed to observe release of hemoglobin as described earlier (Singh et al., 2014). PBS and 0.1% Triton X-100 were used as agents for 0 and 100% hemolysis, respectively.

Phytotoxicity of lipopeptide was checked by treating sterilized *Vigna radiata* (Moong) seeds with different concentrations of A52 lipopeptide (20–60 μg/ml). Lipopeptide was replaced with water as control. Seeds were allowed to germinate and seedlings were maintained at room temperature under moist conditions. Seed germination (%) was recorded for treated and control seeds after 3 days of incubation (Kaur et al., 2016).

### Electron Microscopic Studies

The effect of peptide on cell membrane was determined by scanning electron microscopy (SEM) and transmission electron microscopy (TEM) after treatment with AMPs. For this, test strains *S. aureus* MTCC 1430 and *C. albicans* MTCC 183 were cultured to mid-log phase in respective media. The cells were harvested by centrifugation at 10,000 × g for 10 min, washed thrice with 1X PBS, and re-suspended to adjust an OD_600_ of 0.2. The cell suspension was mixed with 2X and 5X MIC of AMPs and incubated at 37°C overnight. Following the incubation, cells were processed for SEM and TEM as described (Raje et al., 2006). The specimens were completely dried using freeze drier, coated with gold, and visualized under a scanning electron microscope (ZEISS, Germany). For TEM (JEOL, JEM 2100, United States), cells were treated with peptide, washed, and subjected to negative stain 0.1% sodium phosphotungstate (Sigma, United States) for visualization. Effect of A52 lipopeptide on fungal spore was observed through a phase contrast microscope (Nickon H600L, Japan).

### Determination of Fractional Inhibitory Concentration

Fractional inhibitory concentration (FIC) testing was performed to determine the effect of A52 lipopeptide in combination with fluconazole against *C. albicans* MTCC 183. Varying concentrations of fluconazole (0.315–20 μg/ml) and A52 lipopeptide (0.375–12 μg/ml) were used individually or in combination to check the additive effect of antimicrobials. Next, to distinguish between the additive and synergistic effects, we determined the ΣFIC values (Chaturvedi et al., 2011). The interpretation of ΣFIC values was as follows: ≤ 0.5, synergistic; > 0.5 to < 4.0, indifferent (no antagonism), and ≥ 4.0, antagonistic.

### Preparation of Emulgel Formulation

Gel base was prepared by dispersing carbopol 934 (Hi-media, India) in distilled water with constant stirring at moderate speed for overnight. Oil phase of the emulsion was made with cetomacrogol 1000, cetostearyl alcohol, white soft paraffin, propylene glycol in light liquid paraffin (Hi-media, India). Aqueous phase was prepared by dissolving peptides (0.5% w/v) in distilled water. The oily and aqueous phases were separately incubated in a water bath at 60°C. Upon obtaining uniformity, oily phase was added to the aqueous phase with continuous stirring. Pre-heated gel base dispersion of carbopol 934 and vitamin ETPGS (Sigma, United States) were added to the emulsion steadily with continuous stirring to obtain homogenized gel. The temperature of the formulation was lowered to 40°C and isopropyl alcohol was added with continuous stirring. The pH was maintained at 6.8 ± 0.2 using saturated solution of citric acid. *In vitro* emulgel was characterized for its visual appearance under the microscope for the presence of any particle or grittiness. The pH of emulgel was determined by digital pH meter (Contech, India) by dissolving gel in distilled water. Gel extrudability or oozing out from the collapsible tube was determined by physical squeezing from a tube. Gel viscosity was measured using viscometer (Brookfield DV-l Prime, United States).

### Skin Irritation Study

Skin irritation potential of lipopetide based emulgel formulation was evaluated by carrying out skin irritancy test using 8 weeks old BALB/c female mice (Draize et al., 1944). Mice dorsal surface hair was removed without damaging the skin surface about 24 h prior to the experiment. Mice were divided into three groups (*n* = 3): Group I: 20% sodium lauryl sulfate (SLS) solution (positive control); Group II: plain gel (negative control); and Group III: A52 emulgel. The formulation was topically applied to the hairless skin area (approximately 1 cm^2^) and observations were recorded at 24, 48, and 72 h. Thus, the applied sites were observed for dermal reactions such as erythema and edema. The mean erythemal and edemal scores were recorded on the basis of their degree of severity caused by application of formulations as follows: no erythema/edema = 0, slight erythema/edema = 1, moderate erythema/edema = 2, and severe erythema/edema = 3.

### Statistical Analysis

All results were obtained by performing triplicate experiments in three independent replicates and presented as the mean ± SD (standard deviation). Animal experiments were triplicates performed in two independent replicates. Statistical significance among groups (between treated and untreated groups during killing kinetics and treated and positive control groups during hemolysis) were calculated using student’s *t*-test. A *p*-value of < 0.05 was considered significant (^∗^*p* < 0.05, ^∗∗^*p* < 0.005, and ^∗∗∗^*p* < 0.0005).

## Results

### Characterization of Strain A52

**FIGURE 1:**
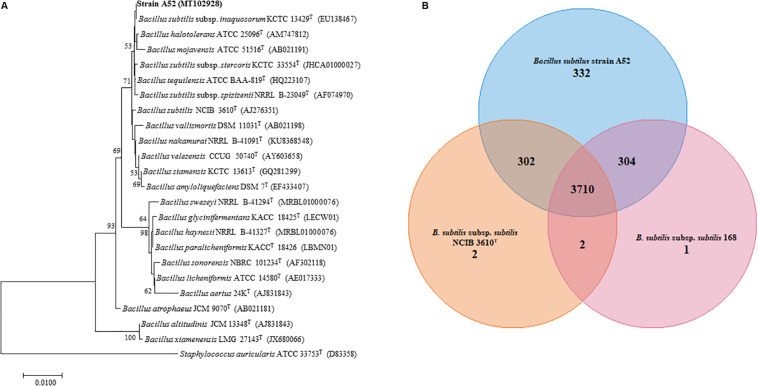
Phylogenetic analysis to determine the taxonomic relation of strain A52. **(A)** Neighbor-joining phylogenetic tree based on 16S rRNA gene sequence of A52 and closely related members of genus *Bacillus*. It was reconstructed by computing evolutionary distances by Tamura-Nei method and expressed in the units of the number of base substitutions per site. The bootstraps values (percentage of 1,000 replicates) are indicated next to branch or nodes. Bar = 0.01nucleotide substitution per site nucleotide. **(B)** Venn diagram showing the number of unique, accessory and core genes of strain A52 along with *B. subtilis* ATCC 6051^T^ and *B. subtilis* subsp. *subtilis* strain 168 created. The number of unique genes of each species is shown in each corresponding circle, while the number of accessory genes is shown in the intersection area and number of core genes which is common for all strains.

A colony on nutrient agar plate showing inhibition activity was isolated and designated as A52. Microscopy examination revealed cells as Gram-positive, rod shaped, motile, and endospore forming. It was catalase positive but methyl red, citrate, and oxidase negative. Optimum growth was observed at 37°C and pH 7. It grew well in the presence of 5% NaCl concentration. Hydrolysis of esculin, starch, gelatin, and tween 20, 40, 60, and 80 was positive. BLAST analysis of strain A52 showed high identity with type species of *B. subtilis* subsp. *inaquosorum* KCTC 13429^T^ (99.93%), *B. tequilensis* KCTC 13622^T^ (99.93%), and *B. subtilis* subsp. *subtilis* NCIB 3610^T^ (99.86%). Neighbor-joining phylogenetic analysis of strain A52 16S rRNA gene resulted in formation of clade with *B. subtilis* subsp. *inaquosorum* KCTC 13429^T^ (Figure 1A), consistently. Nonetheless, the ANI and dDDH values obtained for *B. subtilis* subsp. *subtilis* NCIB 3610^T^ with strain A52 were found to be 98.43 and 86.0%. This higher identity with *B. subtilis* subsp. *subtilis* NCIB 3610^T^ indicates that the strain is pertaining to this subspecies. Similarly, ANI value for the other *B. subtilis* subsp. *spizizenii* TUB-10^T^, *B. subtilis* subsp. *inaquosorum* KCTC 13429^T^, and *B. tequilensis* KCTC 13622^T^ with A52 were found to be 93.07, 93.01, and 91.63%, respectively. Further, the dDDH values for *B. subtilis* subsp. *spizizenii* TUB-10^T^, *B. subtilis* subsp. *inaquosorum* KCTC 13429^T^, and *B. tequilensis* KCTC 13622^T^ with A52 were found to be 50.5, 50.10, and 44.9%, respectively. The 16S rRNA gene and draft genome sequences have been deposited at NCBI with accession numbers MT102928 and JAACYL000000000, respectively. The pangenome analysis revealed a total number of 3,710 core genes (Figure 1B) amongst all the closest relatives of *B. subtilis* strains that exhibited highest identity in dDDH. Maximum unique genes (332) were found in strain A52 in comparison to *B. subtilis* subsp. *subtilis* NCIB 3610^T^ and strain 168.

### Purification and Characterization of A52 Peptides

**FIGURE 2:**
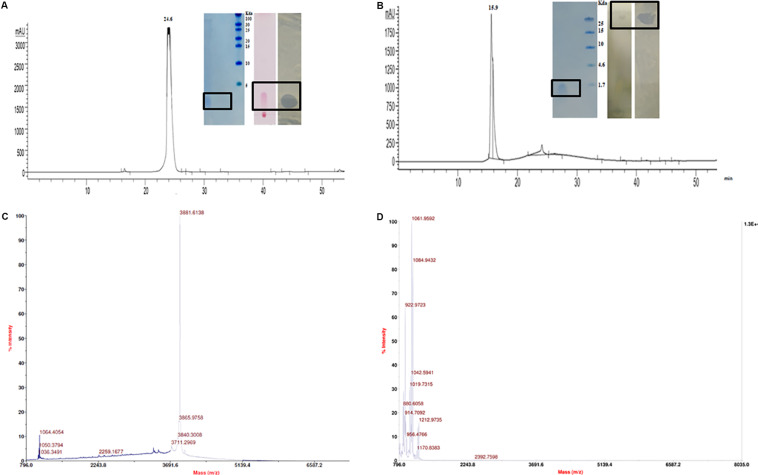
Purification and characterization of antimicrobial peptides. **(A)** RP-HPLC profile of peptide inset showing Tricine–SDS-PAGE of peptide, TLC stained with ninhydrin and bioautography demonstrating a clear inhibition zone against *S. aureus.* **(B)** RP-HPLC profile of lipopeptide inset showing Tricine–SDS-PAGE of lipopeptide, TLC of with Phosphomolybdic acid and bioautography demonstrating a clear inhibition zone against *S. aureus.* **(C)** MALDI analysis of peptide **(D)** MALDI analysis of lipopeptide.

The CFB obtained from 48 h fermented broth of strain A52 in NB showed promising activity against test strains including bacteria and fungi. Antimicrobial substance production (as measured by inhibition zone) was initiated at mid logarithmic phase (1.5 OD_600_) and reached to the highest levels at an OD_600_ of 2.4. Likewise, strain A52 grown in minimal medium containing 1% glucose or sucrose as carbon source yielded the highest inhibition zone of 15 mm against indicator strain, *S. aureus*. Addition of starch or glycerol and peptone as carbon and nitrogen sources showed moderate antimicrobial production (12 mm). Nevertheless, antimicrobial substance production was comparable in NB (13 mm) and the same was used for large scale production (1 L) as optimized medium. In view of multiple antimicrobial production ability by strains of *B. subtilis*, the antimicrobial substance from strain A52 was extracted by two methods including Diaion HP-20 resin (reverse phase chromatography, which capture hydrophobic compounds) and ethyl acetate solvent extraction. Crude extracts obtained in both methods displayed presence of major and minor peaks in reverse phase-HPLC analysis. The major fraction collected at 24.6 min (Figure 2A) from Diaion HP-20 extract yielded a single band corresponding to molecular weight of 3 kDa and showed antimicrobial activity against *S. aureus* MTCC 1430 in in-gel assay (inset Figure 2A). The other peptide collected at RT 15.9 min (Figure 2B) from ethyl acetate extract also showed single band corresponding to 1 kDa. No antimicrobial activity was detected in in-gel assay, but *S. aureus* MTCC 1430 was inhibited in TLC based overlay bioautographic assay (inset Figure 2B). MALDI-MS analysis of purified antimicrobial peptide (peak at RT 24.6 min) displayed a mass of 3881.6 Da (Figure 2C). Similarly, peak at RT 15.9 min yielded strong signals at m/z 1061.9 and 1084.9 Da (Figure 2D) corresponding to protonated peptide ion [M + H]^+^ and its sodium adduct [M + Na]^+^. It also displayed an isomeric form of lipopeptide with m/z 1064.9 (Supplementary Figure S1A). Both peptides analyzed on TLC were stained with ninhydrin. Peptide with 1061.9 Da mass was also stained with phosphomolybdic acid and tested positive in CDA by exhibiting drop collapse.

### Genome Sequence Analysis

**FIGURE 3:**
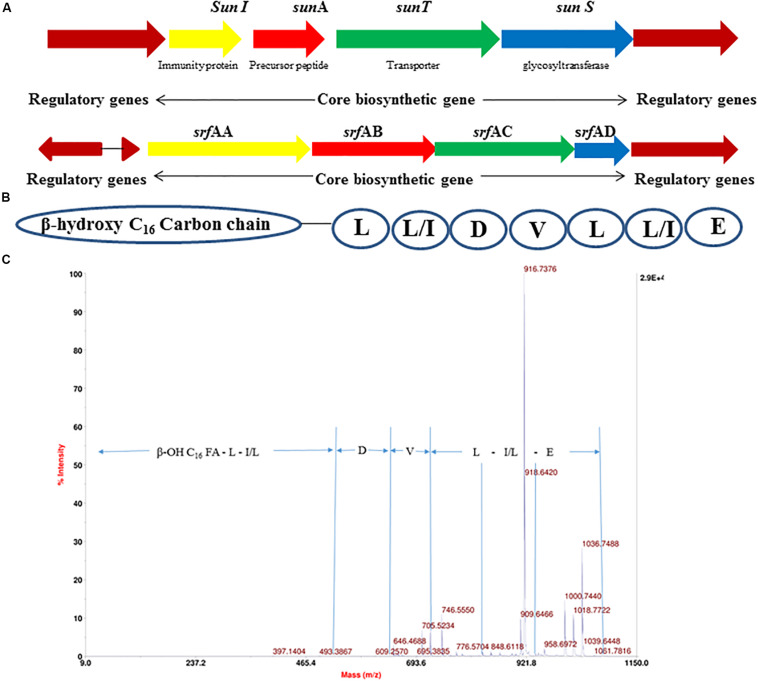
Gene cluster of antimicrobial peptides from strain A52 genome sequence involved in biosynthesis of **(A)** sublancin, **(B)** surfactin, **(C)** fragmentation spectrum of surfactin isoform [M + H]^+^ at m/z 1061.7 Da.

The 4.22 Mb size draft genome was obtained in 59 contigs with an assembly coverage of ∼138X. The G + C content was 43.41 mol%. Analysis of genome sequence using RAST server revealed presence of 4,373 coding sequences (CDS) that contained various gene clusters including those associated with stress and electrolyte homeostasis required for adaptation of marine life, core metabolism, and synthesis of antimicrobial substances. Further, prediction of secondary metabolite biosynthesis gene clusters using antiSMASH identified the presence of eight complete biosynthetic gene clusters involved in synthesis of various antimicrobials secreted by different strains of *B. subtilis* (Table 1). Strikingly, peptide with mass of 3881.6 Da was matched with the mass of an antimicrobial glycocin known as sublancin 168, which showed 100% nucleotide identity with one of the biosynthetic clusters (Figure 3A). The other peptide with mass m/z 1061.9 Da was in agreement to the mass observed for standard surfactin (Supplementary Figure S1B). Consequently, the surfactin encoding non-ribosomal peptide synthatase gene cluster with 82% identity in strain A52 was analyzed in detail (Figure 3B). Furthermore, MS/MS fragmentation pattern resulted in formation of product ion at m/z 705.5, which is presumed to the loss of [Glu + Ile/Leu + Leu + H]^+^, ion at m/z 609.2 [Val + H]^+^, and ion at m/z 493.3 [Asp + H]^+^ from precursor ion m/z 1061.7 Da (Figure 3C). The *de novo* sequence hypothesized for surfactin-like lipopeptide was in alignment with the presence of non-ribosomal synthetases encoding 7 amino acids including Glu^1^-Leu/Ile^2^-Leu^3^-Val^4^-Asp^5^-Leu^6^-Leu/Ile^7^ and a β-OH hexadecane fatty acid chain.

**TABLE 1:** Different secondary metabolite clusters present in strain A52 and its closely related spp. (1) Strain A52, (2) *B. subtilis* subsp. *subtilis* strain 168, (3) *B. subtilis* subsp. *spizizenii* strain TUB-10^T^, (4) *B. subtilis* subsp. *inaquosorum* KTCC 13429^T^, (5) *B. tequilensis* KTCC 13622^T^.

S. No.	Antimicrobial peptides	Class of antimicrobials	1	2	3	4	5	Peptide mass (Da)	References
1	Subtilosin A	Sactipeptide	+	+	+	+	+	3015.5	Babasaki et al., 1985
2	Bacilysin	NRPS dipeptide	+	+	+	+	+	271.1	Phister et al., 2004
3	Subtilin	Lanthipeptide	+	−	+	−	−	3321.7	Parisot et al., 2008
4	Bacillibactin	Siderophore	+	+	+	+	+	881.2	May et al., 2001
5	Sublancin 168	Lanthipeptide	+	+	−	−	−	3880.7*	Oman et al., 2011
6	Bacillaene	Polyene antibiotic	+	+	+	+	−	581.3	Butcher et al., 2007
7	Fengycin	Lipopeptide	+	+	−	+	+	1443.7	Villegas-Escobar et al., 2013
8	Surfactin	Lipopeptide	+	+	+	+	+	1058.6	Villegas-Escobar et al., 2013

*Molecular weight of reduced peptide.

### Minimum Inhibitory Concentration, Killing Kinetics, and FIC

**FIGURE 4:**
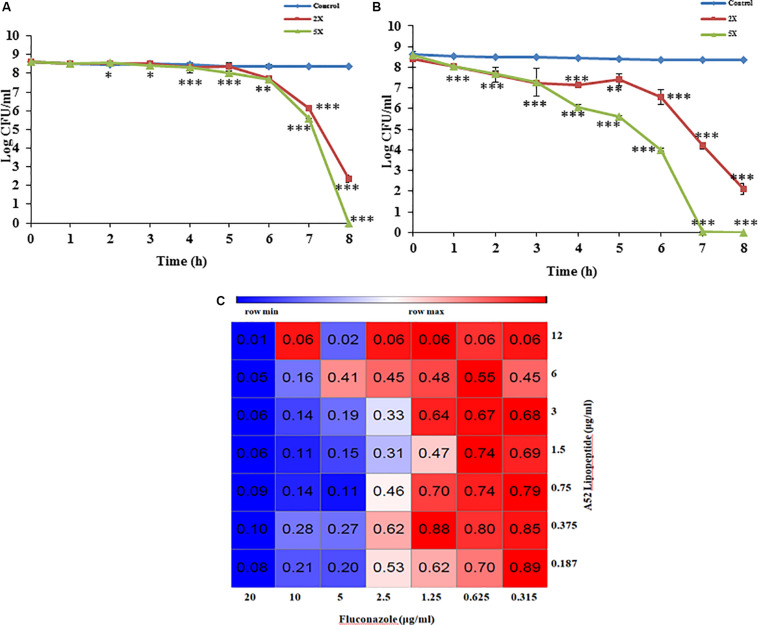
Antimicrobial peptide mediated growth inhibition studies. **(A)** Killing kinetics of sublancin A52 (2X and 5X MIC) against *S. aureus* MTCC 1430. **(B)** Killing kinetics of surfactin-like lipopeptide (2X and 5X MIC) against *S. aureus* MTCC 1430. **(C)** The heat-map showing combination of surfactin-like lipopeptide from A52 and fluconazole inhibitory activity against indicator strain *C. albicans* MTCC 183. Addition lipopeptide (0.75 μg/ml) led to fourfold (2.5 μg/ml) decrease in MIC of fluconazole. The heat-map was generated using the morpheus software. ^∗^*p* < 0.05, ^∗∗^*p* < 0.005, and ^***^p < 0.0005.

Sublancin purified from strain A52 showed activity against Gram-positive bacteria including various clinical strains of *Streptococcus* with MIC concentrations ranging between 2 and 10 μg/ml (Table 2). However, growth of few *Bacillus* species like *B. firmus* (MTCC 488), *B. licheniformis* (MTCC 429), *B. thuringiensis* (MTCC 1953) was not inhibited. No activity was detected against *E. coli* MTCC 1610 or fungal test strains. In contrast, surfactin-like lipopeptide showed broad spectrum antibacterial activity. Also, inhibited all test strains belonging to *Candida* and filamentous fungi (Supplementary Figure S2). The MIC values against bacterial indicator strains were lesser than sublancin (Table 2). Additionally, it was active against fluconazole resistant strains of *Candida* and filamentous fungi with MIC between 12 and 35 μg/ml (Table 2). Bactericidal kinetic studies of sublancin A52 (5X MIC) using cells of *S. aureus* MTCC 1430 revealed reduction of bacterial load completely after 8 h of treatment (Figure 4A) whereas lipopeptide (5X MIC) showed complete killing of *S. aureus* MTCC 1430 within 7 h of treatment (Figure 4B). No reduction in CFU count was noticed for negative controls. A combination of the surfactin-like lipopeptide and fluconazole was found to reduce MIC of fluconazole against *C. albicans* MTCC 183. Remarkably, the treatment in combination led to four times decrease in the MIC of fluconazole in the presence of A52 surfactin-like lipopeptide (Figure 4C), suggesting their combinatorial effect to be synergistic (ΣFIC = 0.375).

**TABLE 2:** MIC values of surfactin-like lipopeptide and sublancin from strain A52 against various test strains of bacteria, yeast, and filamentous fungi.

S. No.	Test organism	Strain number	MIC value (μg/ml)^$^				
			
			Fluconazole*	Amphotericin B*	Ampicillin^†^	Lipopeptide A52	Sublancin^#^
	**Yeast**						
1	*C. albicans*	MTCC 183	20	5	–	12	–
2	*C. albicans*	MTCC 1637	R	5	–	28	–
3	*C. albicans*	MTCC 227	R	5	–	24	–
4	*C. tropicalis*	MTCC 184	R	35	–	12	–
5	*C. glabrata*	MTCC 3019	R	25	–	16	–
6	*C. haemulonii*	MTCC 2766	R	100	–	20	–
7	*C. inconsplicue*	MTCC 1074	50	10	–	16	–
	**Clinical Isolates**						
8	*C. aurius*	470126	R	5	–	26	–
9	*C. rugos*	470141	5	5	–	16	–
10	*C. kefyr*	410004	R	50	–	26	–
11	*C. krusei*	440009	1	5	–	12	–
12	*C. albicans*	400054	R	5	–	16	–
13	*C. tropicalis*	420024	R	50	–	28	–
14	*C. parasilosis*	450030	10	50	–	28	–
15	*C. glabrata*	SKSS ID	R	100	–	16	–
16	*C. haemulonii*	470125	R	50	–	28	–
	**Fungus**						
17	*A. brassicicola*	MTCC 2102	R	50	–	25	–
18	*C. acutatum*	MTCC1037	R	R	–	35	–
19	*F. moniliforme*	MTCC 158	10	50	–	20	–
	**Bacteria**						
20	*S. aureus*	MTCC 1430	–	–	0.25	4	10
21	*B. subtilis*	MTCC 121	–	–	0.50	2	5
22	*B. cereus*	MTCC 430	–	–	0.50	2	5
23	*B. coagulans*	MTCC 492	–	–	0.50	2	8
24	*S. pyogenes*	MTCC 1928	–	–	0.03	2	2
25	*S. anginosus*	MTCC1929	–	–	0.25	4	6
26	*S. oralis*	MTCC 2696	–	–	0.40	4	8
27	*S. mutans*	MTCC 497	–	–	0.40	6	10
28	*M. luteus*	MTCC 106	–	–	0.20	1	2
29	*E. coli*	MTCC 1610	–	–	1.0	6	–

*Antifungal compounds that have no activity against bacteria. ^†^Antibacterial broad spectrum. ^#^Reported as narrow spectrum antibiotic by Paik et al. (1998). ^$^Each test done in triplicate and optical density (OD_600_) of antimicrobial peptide treated wells were compared with uninoculated wells (blank medium control).

### Temperature and Enzyme Sensitivity

Sublancin from strain A52 was found to be stable up to 70°C and lost activity completely after autoclaving. It was also stable between pH 4.0 and 10.0. On the other hand, antimicrobial activity of the surfactin-like lipopeptide from strain A52 was not affected upon exposure to 100°C, but retained 89.0% activity after autoclaving (121°C for 15 min). It was found to be stable between pH 4.0 and 10.0, however, complete loss in activity was noticed at pH 2.0 and 11.0. Studies on tolerance of the peptide to proteolytic enzymes revealed that both peptides were resistant to trypsin and proteinase K treatment (Supplementary Table S1). Additionally, lipopeptide was found to be stable to lipase treatment.

### Microscopic Examination of Pathogens After Peptides Treatment

**FIGURE 5:**
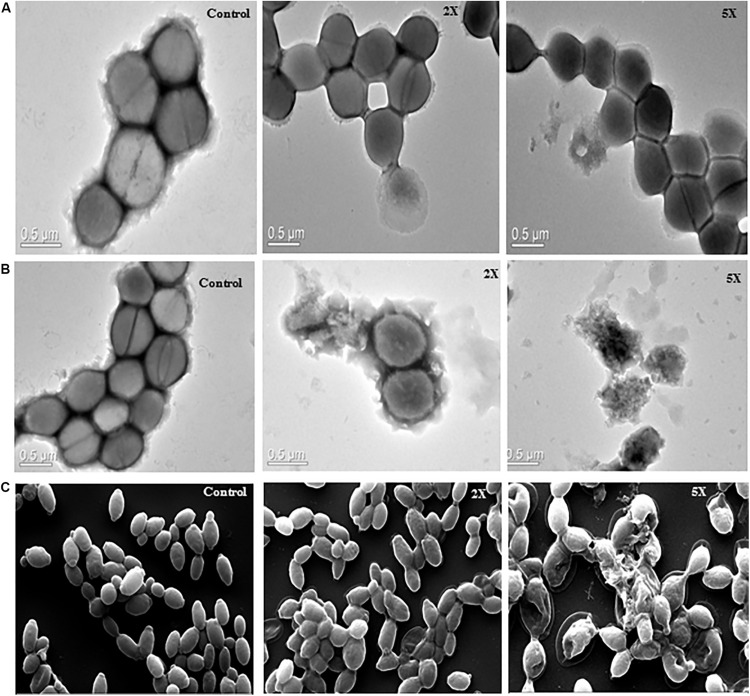
Transmission electron microscopy images **(A)** sublancin A52 treated *S. aureus* MTCC 1430 cells, **(B)** surfactin-like lipopeptide treated *S. aureus* MTCC 1430 cells, **(C)** scanning electron microscopy images of *C. albicans* MTCC 183 (magnification 10000x) after treatment with A52 lipopeptide.

The images of untreated *S. aureus* cells showed smooth cell surface and appeared to be healthy and intact. The sublancin A52 treated cells showed altered morphology and shrinkage in TEM images, whereas the surfactin-like lipopeptide treated cells showed cell wall irregularities such as wall rupturing and cellular lysis (Figures 5A,B). Similarly, SEM images of surfactin-like lipopeptide treated *C. albicans* cells showed structural irregularity with disintegrated outer cell membrane resulting in dispersion of the intracellular contents while the control cells showed complete cell membrane and homogeneous cytoplasm (Figure 5C).

### Hemolysis and Phytotoxicity

**FIGURE 6:**
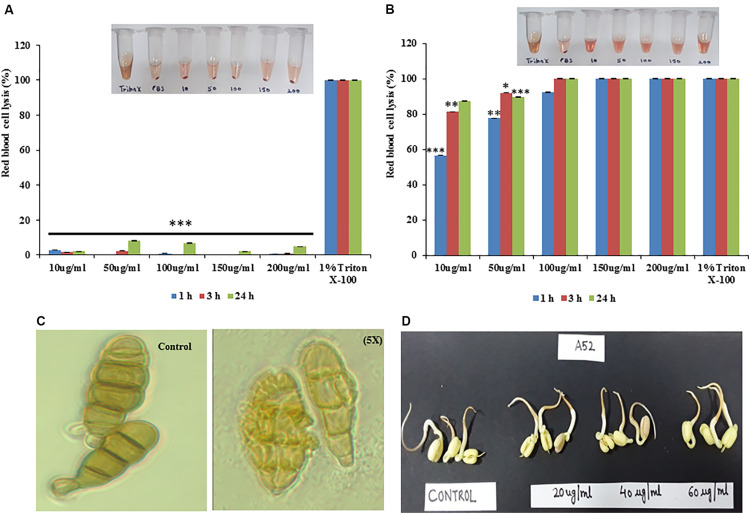
Hemolytic properties of antimicrobial peptides from strain A52 **(A)** sublancin, **(B)** surfactin-like lipopeptide, **(C)** effect of surfactin-like lipopeptide on *A. brassicicola* spore, **(D)** Phytotoxicity of surfactin-like lipopeptide on seeds germination of *Vigna radiata.* ^∗^*p* < 0.05, ^∗∗^*p* < 0.005, and ^***^p < 0.0005.

Sublancin from strain A52 did not show any hemolytic activity even at the 5X MIC concentration (Figure 6A) but surfactin-like lipopeptide demonstrated hemolytic activity at 1X MIC concentration (Figure 6B), which is almost equivalent to the activity shown by 1% Triton X-100. Surfactin-like lipopeptide was also able to lyse the spores formed by phytopathogenic fungus *A. brassicicola* MTCC 2102 (Figure 6C). It did not exhibit phytotoxicity in seed germination assay against *Vigna radiata* (Moong) seeds even at 60 μg/ml concentration. Indeed, there was no difference observed in germination and seedling formation between test and control (Figure 6D).

### Emulgel Formulation and Properties

For preparation of emulgel, 50 mg of surfactin-like lipopeptide was dissolved in water and added to 10 g of emulgel formulation to make a final concentration of 0.5% w/w. The optimized chemical composition (%) used in the preparation of emulgel was propylene glycol (15.0%), cetomacrogol 1000 (2.5%), cetostearyl alcohol (7.5%), white soft paraffin (10.0%), liquid paraffin (2.5%), vitamin E TPGS (5.0%), carbopol 934 (0.5%), isopropyl alcohol (1.0%), and water (55.5%). *In vitro* assessment of emulgel formulation containing surfactin-like lipopeptide exhibited activity against *M. luteus* MTCC 106 and *C. albicans* MTCC 183 (Supplementary Figure S3). Individual testing of the above compounds at employed concentrations did not show antimicrobial activity against test strains. The emulgel was opaque and creamish in its appearance. The pH of emulgel was found to be 6.8 ± 0.2 and showed good extrudability. The viscosity of emulgel containing 0.5% w/w of carbopol was found to be 1451cP.

### Skin Irritation Study

**FIGURE 7:**
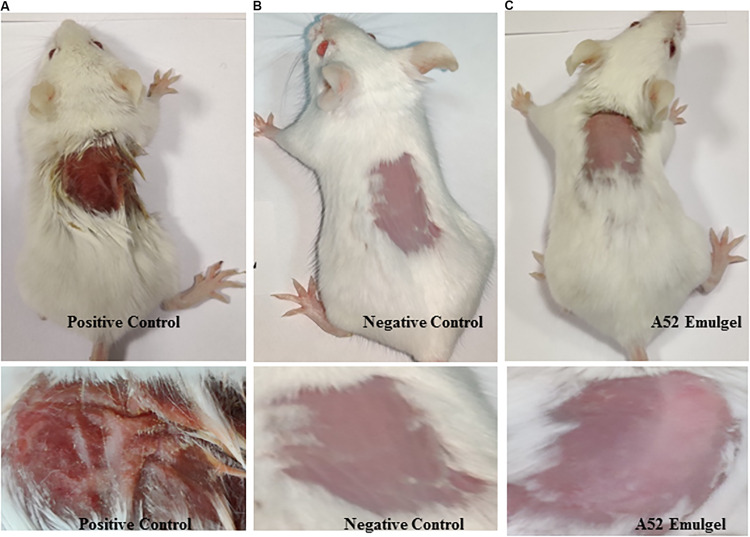
Skin irritation study results at 72 h **(A)** positive control, 20% SLS treated, **(B)** negative control, plain gel, and **(C)** treated with emulgel containing surfactin-like lipopeptide.

Results of skin irritancy of surfactin-like lipopeptide containing emulgel formulation determined using Draize test are summarized in Supplementary Table S2. The emulgel did not show any type of irritation as observed at time intervals of 24, 48, and 72 h after application. Dryness, hardening, and redness of skin was noticed at the site of SLS application which is known to cause irritation (Figure 7A). No effect was detected in negative control (Figure 7B). The results of emulgel formulation application on skin was found to be similar with negative control (Figure 7C). There were no irritation symptoms observed even after 72 h upon application.

## Discussion

Increased resistance to antibiotics has resulted in difficulty to eliminate nosocomial and chronic infections. In the quest to develop novel antimicrobial agents, the marine ecosystem is favored environmental niche owing to the presence of diverse antimicrobial substances (Desjardine et al., 2007; Baindara et al., 2013; Ponnappan et al., 2015; Agrawal et al., 2017; Gupta et al., 2019). Therefore, in this study, we have explored a marine sediment bacterial isolate for antimicrobial production ability. Strain A52 was identified as a member of the genus *Bacillus*, taxonomically related to species *B. subtilis* (Tareq et al., 2014) and *B. tequilensis* (Rani et al., 2016). The ANI and dDDH of A52 genome sequence showed highest identity with *B. subtilis* subsp. *subtilis* NCIB 3610^T^ and followed by strain 168 (98.4 and 85.9%, respectively). Strain A52 mainly differed with both strains in number of unique genes, mostly involved in adaptation to marine environment. RAST, and bioinformatics analyses of genome sequence revealed the ability of strain A52 to produce multiple antimicrobials. Thus, strain A52 was grown in the presence of different carbon and nitrogen sources to test the possibility of multiple antimicrobials production as mentioned in Table 1. Since crude extract inhibited the growth of indicator strains pertaining to Gram-positive, Gram-negative bacteria, and *Candida*, we have analyzed the extract (including extracts from NB and MM with 1% glucose) on HPLC and detected the presence of two compounds with different spectrum of antimicrobial activity. Both purified compounds analyzed on TLC were stained with ninhydrin, indicating possible peptide nature of compounds. The low molecular weight compound stained with phosphomolybdic acid yielding a bluish black band, exposed association of a lipid group with the peptide. Lipopeptide presence was also confirmed with the increased surface diameter in collapse drop assay.

The validated mass of 3881 Da as detected for peptide from strain A52 is in alignment with the mass 3880.78 Da of reduced sublancin, a glycocin antimicrobial peptide produced by closest relative *B. subtilis* strain 168 (Oman et al., 2011; Wang et al., 2017). The reduced nature of peptide in this study is assumed to be due to the presence of acentonitrile in 0.1% TFA (Erlandsson and Hallbrink, 2005) as the peptide was not freeze dried to remove the solvent upon HPLC purification. The minor difference in mass may be due to instrument error (50 ppm deviation). Since sublancin belongs to the class of glycocins, which contains an unusual and essential glucosylated Cys residue (Gonzalo et al., 2014; Ji et al., 2015; Wang et al., 2017; Wu et al., 2019), we have verified the presence of sugar residue using α-naphthol reagent and confirmed the peptide as sublancin. Furthermore, the peptide inhibited the growth of only Gram-positive bacteria as described for sublancin (Oman et al., 2011). Number of Gram-positive test strains used for antimicrobial assay showed the ability of sublancin to kill various species of the genus *Bacillus* with few exceptions owing to the fact that sensitivity of various strains to the same bacteriocin is highly variable (Rasch and Knøchel, 1998). Sublancin is also reported to inhibit growth of methicillin resistant *Staphylococcus aureus* (MRSA) and showed protective ability in mice infection studies (Wang et al., 2017). TEM imaging of *S. aureus* cells treated with sublancin from A52 provided clear evidence that it possesses bactericidal activity without cell lysis (Figure 5A), which is in accordance with recently studied mode of action of sublancin that reported negative effect on DNA replication, transcription, and translation (Wu et al., 2019). The thermal stability, broad pH range, and resistance to proteinase K and trypsin are in accordance with results observed earlier (Ji et al., 2015).

Likewise, the antiSMASH analysis for strain A52 genome sequence highlighted presence of a NRPS encoding genes with 82% nucleotide sequence identity to biosynthetic gene cluster involved in surfactin biosynthesis. As shown in mass spectrometry lipopeptide has a molecular weight of 1061 Da, which falls within the range of observed mass for surfactants. Combining the data from genes encoding NRPS clusters and mass obtained, we hypothesized that the lipopeptide consists of 7 amino acids as shown by the presence of specific synthetases which are commonly found in surfactin biosynthesis (Ma et al., 2016; Chen et al., 2017). The fatty acid moiety was identified as β-OH palmitic acid chain that has not been reported in surfactins produced by *B. subtilis* strains. Unlike other surfactins, the lipopeptide was found to be highly thermostable as it retained activity even after autoclaving. No reduction in activity was observed upon incubation with proteases or lipase. These results are in line with earlier reported lipopeptide properties in literature (Sharma et al., 2014; Jha et al., 2016; Chen et al., 2017). Strikingly, surfactin-like lipopeptide produced by strain A52 showed broad spectrum antimicrobial activity against bacteria at low concentrations. Though inhibition of phytopathogenic fungi required higher concentrations, it was lesser than 25 μM that is generally employed to screen antifungal peptides and lipopeptides (Mircus et al., 2015). Earlier reports show that antimicrobial spectrum of lipopeptides were purely dependent on the length of their fatty acid moiety (Strieker and Marahiel, 2009; Mandal et al., 2013; Meir et al., 2017). Thus, it is pertinent to mention that the presence of different fatty acid resulted in broad spectrum antibacterial, anticandidial, and antifungal activities. SEM and TEM imaging of surfactin-like lipopeptide treated cells showed disruption of membrane due to possible pore formation resulting in scattering of cellular contents in their surroundings (Figure 5). This membrane lysis mechanism of action of lipopeptides makes the pathogens highly susceptible by decreasing the ability to develop resistance (Mangoni and Shai, 2011). In fact, surfactant-like lipopeptide was able to inhibit fluconazole resistant *Candida* by lowering the MIC of fluconazole in a synergistic combination. These results suggest that combined usage of the active compound with existing drugs shall contribute toward lowering the adverse effects associated with antifungal agents, while maintaining the effectiveness of the drug.

For commercial application of biologically active compounds, it is imperative to explore their toxigenic potential for the relevant applications. Though lipopeptides cause hemolysis (Inès and Dhouha, 2015; Greber et al., 2018; Ramachandran et al., 2018), surfactin-like lipopeptide in this study showed promising potential for topical applications as evident from the emulgel experiment. The gel showed antimicrobial activity and did not show any skin irritation effect during experiment with BALB/c mice. Further, it was explored for phytotoxicity, where it showed no adverse effects in the seed germination process. Thus it may be developed as a crop-protective agent for use in agriculture field, but only upon detailed experiments, which are beyond the scope of this study. Considering broad activity with low MIC values and thermos-stability, surfactin-like lipopeptide secreted by strain A52 was found to be an appealing candidate for potential application as a topical therapeutic agent.

## Conclusion

A novel broad spectrum antifungal lipopeptide has been reported from *B. subtilis* strain A52 isolated from marine sediment. It is simultaneously expressed with a well-known bacteriocin sublancin. The 1061.9 Da surfactin-like lipopeptide is highly effective against a diverse array of human and plant pathogenic fungi and has a broad spectrum antibacterial activity. Essentially, surfactin-like lipopeptide has shown synergistic activity with contemporary antifungal agent fluconazole, suggesting its promising role as a topical therapeutic agent.

## Data Availability Statement

The datasets generated for this study can be found in the genome sequence accession no. JAACYL000000000 and 16S rRNA gene sequence accession no. MT102928.

## Ethics Statement

The animal study was reviewed and approved by the Institutional Animal Ethics Committee, Committee for the Purpose of Control and Supervision of Experiments on Animals approval no. 18/08.

## Author Contributions

DS, SSS, NK, and SK designed experiments. DS, SSS, PB, SS, PBP, NK, and SK performed experiments. DS, SSS, VG, PBP, and SK analyzed data. DS, SSS, VG, and SK prepared the manuscript.

## Conflict of Interest

The authors declare that the research was conducted in the absence of any commercial or financial relationships that could be construed as a potential conflict of interest.

## Footnotes

1 http://ggdc.dsmz.de/distcalc2.php

2 https://iicb.res.in/bpga/index.html
