# Supplementary material for: Surfactin Like Broad Spectrum Antimicrobial Lipopeptide Co-produced With Sublancin From Bacillus subtilis Strain A52: Dual Reservoir of Bioactives
Source: Front Microbiol. 2020 Jun 11;11:1167. doi: 10.3389/fmicb.2020.01167 (PMC7300217; doi:10.3389/fmicb.2020.01167)

Surfactin like broad spectrum antimicrobial lipopeptide co-produced with sublancin from *Bacillus subtilis* strain A52: Dual reservoir of bioactives

Deepika Sharma[1], Shelley Sardul Singh[1], Piyush Baindara[1], Shikha Sharma[1], Neeraj Khatri[1], Vishakha Grover[2], Prabhu B. Patil[1], Suresh Korpole[1*]

1. CSIR-Institute of Microbial Technology, Sector 39A, Chandigarh, India.
2. Dr. Harvansh Singh Judge Institute of Dental Sciences and Hospital, Panjab University, Chandigarh, India.

*Correspondence: Suresh Korpole, CSIR-Institute of Microbial Technology, Chandigarh-160036, India, email: suresh@imtech.res.in

Supplementary Table S1. Effect of temperature, pH and proteases on sublancin like peptide and lipopeptide from strain A52.

Treatment	Reaction Duration/ Condition	Residual activity (%)	
		Peptide	Lipopeptide
Temperature (ºC)			
37	1h	100	100
50	1h	100	100
60	1h	100	100
70	1h	100	100
80	1h	80.3	100
100	1h	53.3	100
121	15 min (15 lbs)	0.0	89.0
pH			
2.0	4h/ 25°C	Nil	Nil
4.0	4h/ 25°C	86.6	100
6.0	4h/ 25°C	100	100
7.0	4h/ 25°C	100	100
8.0	4h/ 25°C	100	100
10.0	4h/ 25°C	93.3	100
11.0	4h/ 25°C	Nil	80
12.0	4h/ 25°C	Nil	Nil
Enzymes			
Proteinase k	6h/ 37°C	93.3	100
Trypsin	6h/ 37°C	100	100
Lipase	6h/ 37°C	ND	100

ND = Not determined

Supplementary Table S2. Mean erythemal score observed at end of 24, 48 and 72 h after application of lipopeptide containing emulgel.

Test groups	Erythema score (n=3)			
	0 h	24h	48h	72h
Negative Control	0	0	0	0
Positive Control	0	3	3	3
A52 Emulgel	0	0	0	0

Supplementary Figure S1. Mass determination of lipopeptide and surfactin by MALDI-MS. A) Lipopeptide isoform ion at m/z 1064.5 Da and its sodium adduct m/z 1087.5 Da from strain A52. B) Standard surfactin ions (m/z 1035.22 Da) and sodium adduct (m/z 1057.62 Da).

A)

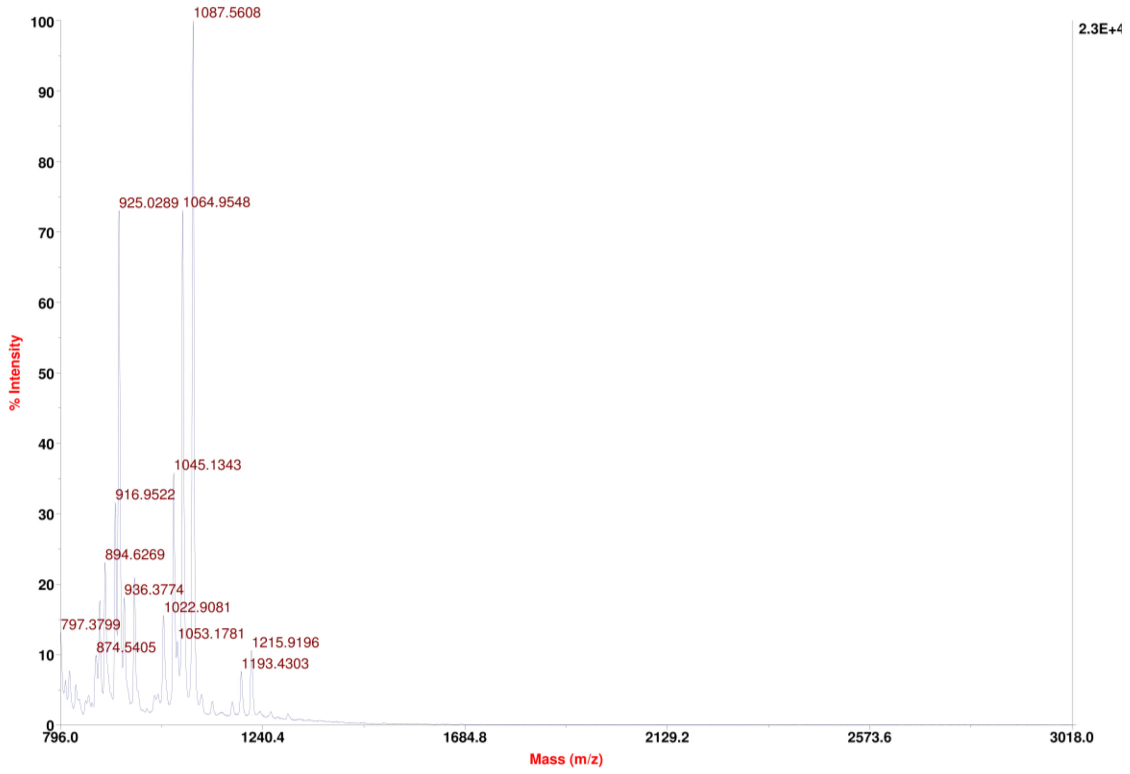

B)

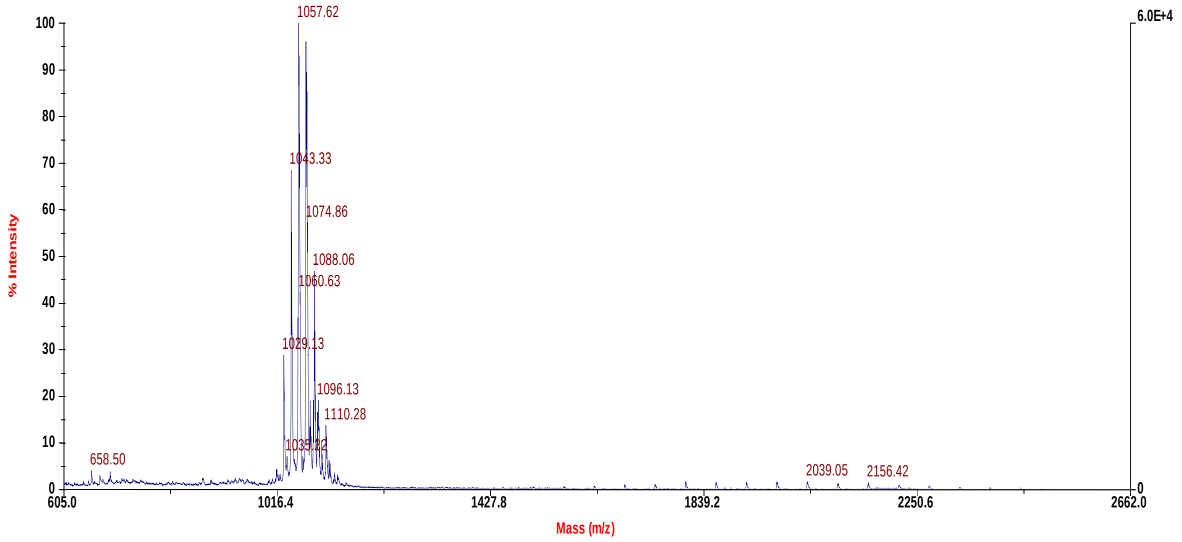

Supplementary Figure S2. Antifungal activity of lipopeptide from strain A52 against (a) *A. brassicicola* MTCC 2102 (b) *C. acutatum* MTCC 1037.

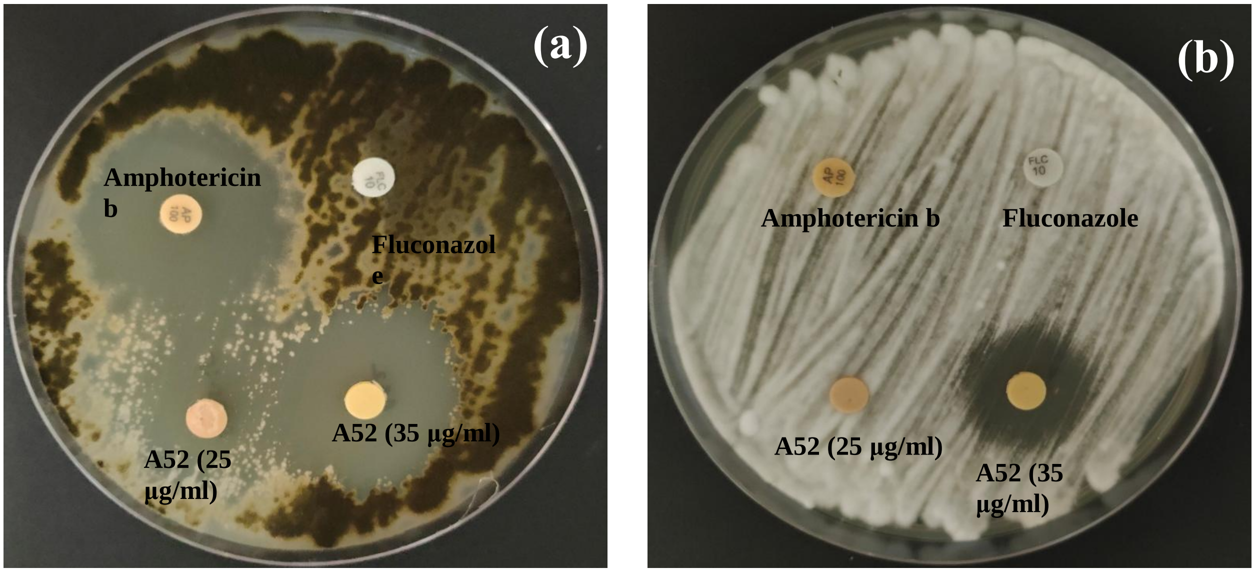

Supplementary Figure S3. Determination of antimicrobial activity of emulgel prepared using lipopeptide against (a) *M. luteus* MTCC 106 (b) *C. albicans* MTCC 183.

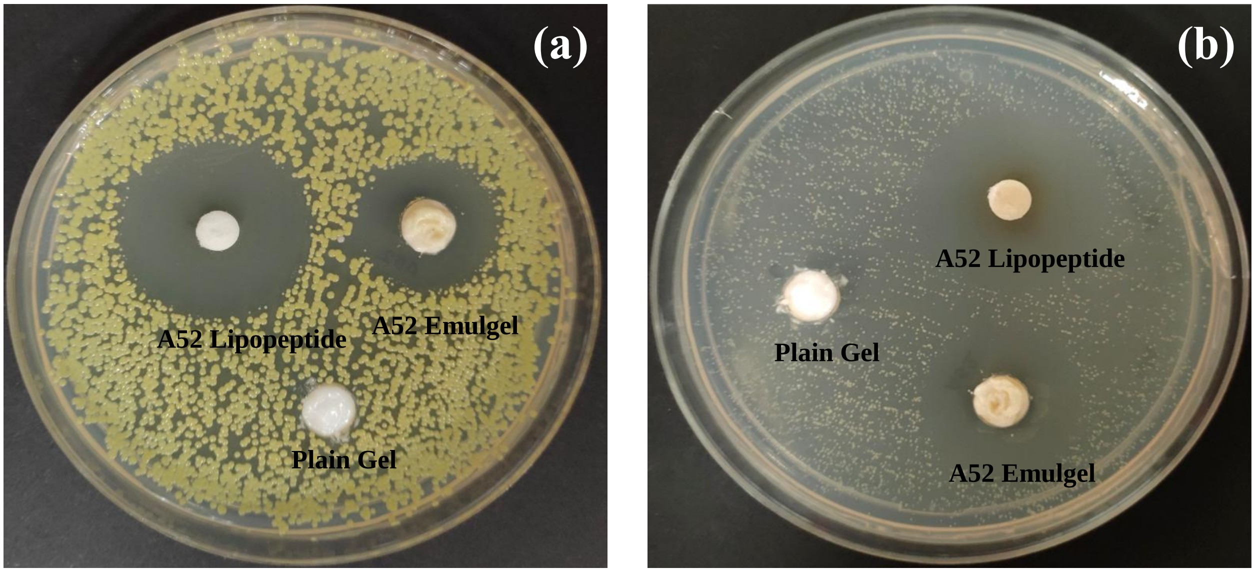

Supplement: Supplementary file 1 [file Data_Sheet_1.pdf]
